# Graves’ disease following hypothyroidism due to Hashimoto’s thyroiditis in a black South African lady: a case report

**DOI:** 10.11604/pamj.2019.32.186.18713

**Published:** 2019-04-17

**Authors:** Chukwuma Ekpebegh, Khaled Elmezughi, Lungiswa Mtingi

**Affiliations:** 1 Department of Internal Medicine, Walter Sisulu University, Mthatha, South Africa

**Keywords:** Graves´ disease, hyperthyroidism, Hashimoto´s thyroiditis, hypothyroidism, autoimmune thyroid disease, South Africa

## Abstract

Graves' disease and Hashimoto's thyroiditis are autoimmune thyroid disorders with distinct pathological and histopathological features. The conversion from Hashimoto's thyroiditis to Graves' disease has been rarely reported throughout the world with no reports in the African race to our knowledge. We here report an African lady who was initially diagnosed with primary hypothyroidism following Hashimoto's disease but later became thyrotoxic due to Graves' disease.

## Introduction

There are few reports in the literature of hyperthyroidism after an initial period of hypothyroidism [1-4]. These have been mainly in Asian and Western populations. It is important to identify the rare patients who develop hyperthyroidism after initial treatment for hypothyroidism to enable prompt discontinuation of thyroxine and institution of appropriate treatment for hyperthyroidism. We found no reports in the English literature of hyperthyroidism following hypothyroidism in an African patient. We herein, report on a middle aged black South African female, who was initially diagnosed with hypothyroidism due to Hashimoto's thyroiditis but later developed Graves' disease after 5 years of thyroxine replacement.

## Patient and observation

A 54-year-old black South African female from the Eastern Cape Province was initially diagnosed in 2011 with primary hypothyroidism secondary to Hashimoto's thyroiditis when she presented with progressive weight gain, constipation and cold intolerance. She had no goiter. Laboratory test results at diagnosis revealed extremely low thyroid hormone levels: serum free T4 of 1.3 pmol/L (12-22) and free T3 of < 0.4 pmol/L (2.8-7.1). This was accompanied with markedly elevated TSH of 86.71 miu/L (0.27-4.2). Thyroid peroxidase antibody level was elevated at 153 iu/ml (0-35). She was subsequently started on thyroxine replacement and the dose was gradually increased to150 mcg daily.

In June 2016, she presented with history of palpitations, heat intolerance, tremors, exertional dyspnoea and bilateral pitting oedema. She had sinus tachycardia of 135 beats per minute with elevated blood pressure of 150/105 mmHg. Thyroid function tests (TFT) at presentation in June 2016 showed markedly elevated free T4 (72.2 pmol/L) and free T3 (30.8 pmol/L) with suppressed TSH of < 0.01 miu/L. She was diagnosed with a thyrotoxic heart failure and thyroxine replacement was stopped. She also received furosemide 40 mg BD, bisoprolol 5mg daily, enalapril 5 mg BD and was later commenced on carbimazole 20 mg daily when her thyrotoxicosis persisted. Subsequently Graves' disease was confirmed by the finding of elevated TSH receptor antibody levels of 6.83 u/L (< 1.75). The TFT results in July 2016 revealed free T4 and free T3 of 20.6 pmol/L and 6.4 pmol/L respectively with suppressed TSH < 0.01 miu/L.

Carbimazole was stopped in July 2018 when follow up biochemistry showed frank hypothyroidism: low serum free T4 of 10.1 pmol/L (12-22) and elevated TSH of 13.9 miu/L (0.27-4.2). She was biochemically euthyroid on follow up visits of September 2018: serum free T4 of 14.9 pmol/L (12-22) and TSH of 2.35 miu/L (0.27-4.2) and February 2019: serum free T4 of 12.3 pmol/L (12-22) and TSH of 0.39 miu/L (0.27-4.2). She has neither been treated with carbimazole nor thyroxine since July 2018. She however remains on antihypertensive medications.

### Consent

Written informed consent for this case report was obtained from the patient. A copy of the written consent is available for review by the editorial board on request.

## Discussion

Our patient initially had clinical and laboratory features of hypothyroidism due to Hashimoto's thyroiditis. When she became thyrotoxic and presented in heart failure after 5 years of 150 mcg daily thyroxine replacement for hypothyroidism, the initial thinking was that of thyroxine induced thyrotoxic heart failure. However, with the finding of elevated serum TSH receptor antibody titers and persistent thyrotoxicosis despite withdrawal of thyroxine, her diagnosis was revised from hypothyroidism due to Hashimoto's to hyperthyroidism due to Graves' disease. Chronic hypothyroidism with inadequate thyroxine replacement can result in pituitary hyperplasia and eventual thyrotoxicosis secondary to an autonomous secretion of TSH [5]. This is however, unlikely in our patient as an elevated and not suppressed TSH would be expected.

Although the transition from thyrotoxicosis to hypothyroidism is rare, it occurs in the setting of Hashitoxicosis where following thyroid follicular cells destruction an initial phase of thyrotoxicosis due to leakage of preformed thyroid hormones is followed by permanent hypothyroidism [6]. Thyrotoxicosis following hypothyroidism is also rare and is often described in Asian and western populations [1-4]. This scenario has been described predominantly in middle aged females [7]. It may occur up to 20 years after an initial period of hypothyroidism [8]. Our case is similar to other reports in literature in being a middle-aged female, the exception is that she is of black race and we have not found reports describing it in the black ethnicity in the English language literature. The factors that have been associated with the transition from hypothyroid Hashimoto's to Graves' disease include thyroxine treatment which may promote the elaboration of TSH receptor stimulating antibodies [1]. Treatment with immunomodulatory agents such as interferon and alemtuzumab have also been implicated in the switch from hypothyroidism to thyrotoxicosis [9, 10]. Our patient, however, neither received interferon nor alemtuzumab. She expectedly received thyroxine replacement for chronic primary hypothyroidism.

In all reports of transition from thyrotoxicosis to hypothyroidism and vice versa, it has been in the setting of autoimmune thyroid disease. The postulated mechanism includes a switch from TSH receptor blocking to TSH receptor stimulating antibodies [11]. Indeed, circulating levels of TSH receptor inhibiting and stimulating antibodies have been reported in the same patient [12]. The clinical manifestation will be determined by which antibody predominates and this may vary with time with the same patient clinically manifesting as Hashimoto's thyroiditis or Graves' disease [13]. Alternating episodes of hypothyroidism and hyperthyroidism may be ultimately stopped with thyroidectomy or radioactive ablation of the thyroid [8, 12].

This case report is not without limitations; we did not test our patient for TSH receptor antibodies when she was diagnosed with hypothyroidism as it is not the standard of care. Our practice is to perform thyroid peroxidase antibody testing in primary hypothyroidism when Hashimoto's is suspected. Thyroid peroxidase antibody; which is positive in 90-95% of patients with Hashimoto's is the preferred test in autoimmune primary hypothyroidism [14, 15]. We also did not perform radionuclide imaging in our patient when she became thyrotoxic to demonstrate the expected pattern of diffusely increased uptake in Graves' disease because of the limited resources in our setting. We are aware that TSH receptor antibodies may be falsely positive in patients with thyrotoxicosis not due to Graves' disease [13]. However, we do not expect this to be the case in our patient.

## Conclusion

In conclusion, our case extends the rare reports of Graves's disease following hypothyroidism to include the black African population.

## Competing interests

The authors declare no competing interests.
